# Communication strategies to help reduce the prevalence of non-communicable diseases: Proceedings from the inaugural IFIC Foundation Global Diet and Physical Activity Communications Summit

**DOI:** 10.1111/j.1753-4887.2012.00480.x

**Published:** 2012-04-26

**Authors:** Madelyn H Fernstrom, Kimberly A Reed, Elizabeth B Rahavi, Carrie C Dooher

**Affiliations:** 1 University of Pittsburgh Medical CenterPittsburgh, PA, USA; 2 International Food Information Council FoundationWashington, DC, USA; 3 Ogilvy WashingtonWashington, DC, USA

**Keywords:** behavior, communication, energy balance, messages, non-communicable disease

## Abstract

Non-communicable diseases (NCDs), which include cardiovascular disease, cancer, and diabetes, all of which are associated with the common risk factors of poor diet and insufficient physical activity, caused 63% of all deaths globally in 2008. The increasing discussion of global NCDs, including at the 2011 United Nations General Assembly High-level Meeting on the Prevention and Control of Non-communicable Diseases, and a request for multi-stakeholder engagement, prompted the International Food Information Council Foundation to sponsor the Global Diet and Physical Activity Communications Summit: “Insights to Motivate Healthful, Active Lifestyles” on September 19, 2011, in New York City. The Summit brought together a diverse group of stakeholders, representing 34 nations from governments; communication, health, nutrition, and fitness professions; civil society; nonprofits; academia; and the private sector. The Summit provided expert insights and best practices for the use of science-based, behavior-focused communications to motivate individuals to achieve healthful, active lifestyles, with the goal of reducing the prevalence of NCDs. Presented here are some of the highlights and key findings from the Summit.

## Introduction

Non-communicable diseases (NCDs) include the “big four”– cardiovascular disease, cancer, diabetes, and chronic respiratory disease. According to the World Health Organization (WHO), NCDs were responsible for 63% of all deaths globally in 2008, with more than 80% occurring in developing countries, and were largely attributed to poor diet, insufficient physical activity, tobacco use, and harmful use of alcohol.^1^ In order to increase public awareness of NCDs and support NCD prevention and control efforts, the United Nations (UN), WHO, Heads of State, Health Ministers, and other government officials have called for multi-sectoral action and multi-stakeholder engagement.^2^

In response to this invitation, the International Food Information Council (IFIC) Foundation, a nonprofit educational organization, with the mission of effectively communicating science-based information on health, nutrition, and food safety to the public, hosted the Global Diet and Physical Activity Communications Summit: “Insights to Motivate Healthful, Active Lifestyles,” on September 19, 2011, in New York City. Civil society organizations, including those that are a part of the NCD Alliance, of which the IFIC Foundation is a common interest group member, are expected to play a key role inensuring that NCDs remain at the fore of local, national, and international health agendas.^2^ The Summit brought together a diverse group of stakeholders to address the role of health communications in reducing the prevalence of NCDs, with a focus on those NCDs associated with the common risk factors of poor diet and physical inactivity. The stakeholders represented a total of 34 nations from the following fields: governments; communications, health, nutrition, and fitness professions; civil society; nonprofits; academia; and the private sector.

## Keynote address of the us surgeon general

In the keynote address, US Surgeon General Regina M. Benjamin indicated that one of the most important steps communicators can take for global health is to provide clear, simple information based on the latest science. Across the globe, prevention is the foundation of public health systems. A comprehensive, holistic approach that provides a solid infrastructure and includes national, state, and local governments; industry; corporations; celebrities; communities; families; and individuals is vital for success.

The United States is leading by example with recent initiatives, such as the release of the *Dietary Guidelines for Americans*, 2010^3^; the US Department of Agriculture's (USDA) new MyPlate icon (http://www.choosemyplate.gov); the First Lady's *Let's Move!* program (http://www.letsmove.org); and *The Surgeon General's 2010 Vision for a Healthy and Fit Nation*.^4^ Each of these efforts provides concrete steps to move the nation from a paradigm of treating sickness and disease to one of prevention. In 2011, the National Prevention, Health Promotion, and Public Health Council, which is chaired by the US Surgeon General and focuses on national wellness, health promotion, and public health goals, released its inaugural *National Prevention and Health Promotion Strategy*.^5^ The strategy is designed to improve the health and quality of life of individuals, families, and communities by setting goals, actions, and timelines; recommending improvements; and prioritizing evidence-based policy and program interventions.

## Rising incidence of non-communicable diseases

John Milner, of the National Cancer Institute, pointed out that until recently, infectious disease has been the focal point of health communications, requiring treatment and preventive measures on a global level, primarily in developing countries. In recent years, however, the incidence of NCDs has increased dramatically and the focus has shifted.

Primary questions remain that must be answered when communicating recommendations to the public about lifestyle changes that decrease the risk of NCDs, such as: “What type of change is needed?”“How much?” and “When?” While it is clear that lifestyle change interventions should begin early, perhaps even in utero, the most effective timing and delivery mechanisms are unknown.

Recognizing the severity of social, economic, and health consequences resulting from the increasing incidence of NCDs, the UN, during the 66^th^ session of the UN General Assembly, took the historical step of hosting the High-level Meeting on the Prevention and Control of NCDs, on September 19–20, 2011, in New York City. This type of health-related meeting has occurred only once before in the UN's history – the 2001 High-level Meeting on HIV/AIDS. As part of the 2011 High-level Meeting, Heads of State and Government from around the world met at the UN and unanimously adopted a Political Declaration to stand united in a global fight against NCDs.^6^

According to Hugh Dugan, of the US Mission to the UN, the UN Political Declaration is a political tool for progress that highlights the global burden of NCDs and the need for prevention. The Declaration recommends ways to implement and monitor prevention strategies and programs and also suggests actions to address the specific challenges facing developing countries. Also referred to as “lifestyle diseases,” NCDs are often considered to be byproducts of economic development. They are, however, becoming more common in developing countries and could impede further economic development.

The success of the UN's efforts depends upon civil society delivering and implementing health messages. The goals of the UN Political Declaration must be supported by accountability and monitoring mechanisms, regular progress reviews, and availability of the necessary international and domestic resources. The UN is expected to evaluate the progress of the measures and interventions outlined in the Political Declaration in 2014.^6^

An important caveat raised by Peter Greenwald, of the National Cancer Institute, for those developing and implementing messages, is that one size does not fit all. Basic principles of preventive measures can be applied to promote a healthful lifestyle, but susceptibility to their effects will vary depending upon a variety of factors, including the demographic targeted and individuals' biological makeup.

The American Institute for Cancer Research has developed a list of the most important small changes individuals can make to reduce the risk of cancer, focusing primarily on diet and exercise. While each of these recommendations should be a goal for every person, not everyone will derive equal benefit.^7^

As the paradigm for effectively reducing the incidence of NCDs shifts from treatment to prevention, it is vital to keep in mind the following guiding principle: in order to communicate and promote public health, strategies must be predictive, personalized, preemptive, and participatory.

## Cost of non-communicable diseases

NCDs are a significant public health concern that strains global economies. Jeffrey L. Sturchio, of Johns Hopkins University, discussed the anticipated economic costs associated with the rise of NCDs over the next 20 years, as well as the cost of effective ways, or “best buys,” to intervene. *The Global Burden of Non-communicable Diseases*, a report developed by the World Economic Forum and the Harvard School of Public Health, projected that the five leading NCDs (cardiovascular disease, cancer, diabetes, chronic respiratory disease, and mental illness) could cost $47 trillion by the year 2030.^8^ In a complementary analysis, a WHO report, *Scaling Up Action Against Noncommunicable Diseases: How Much Will it Cost?*,^9^ estimated the costs of scaling up a core intervention package in low- and middle-income countries. It is estimated that if rates of NCDs continue at the current pace over the next 20 years, the cumulative output loss will far outweigh those of intervention programs – $47 trillion compared to $11.4 billion per year for the 42 countries in which 90% of NCDs are reported to occur. On a per-person basis, the annual cost of intervention ranges from $1 to $3. Possible low-cost strategies could include integrating diet and nutrition information with other health programs and interventions, such as food security.

Sharing successful strategies among nations will foster international cooperation, and promote healthful behavior and healthful choices as populations around the world struggle to combat the rising incidence of NCDs.

## Understanding consumer behavior: progressing toward optimal health

Obesity is a major contributor to NCDs. Initiating and sustaining behavior change related to diet and physical activity is a major challenge facing health professionals, policymakers, and researchers worldwide in their efforts to reverse global obesity trends. Gathering insights on consumer attitudes and perceptions that affect their behavior, as well as establishing partnerships within communities to influence healthful behavior change, are critical steps.

Despina Spanou, of the European Commission, European Union (EU), addressed the unique framework and accomplishments of the European Commission's *European Union Platform on Diet, Physical Activity and Health*, which was established in 2005 and has coordinated the actions of the 27 countries in the EU.^10^

In 2007, following the platform's launch, the Commission of European Communities released a consumer- and behavior-focused policy, *A Strategy for Europe on Nutrition, Overweight and Obesity-related Health Issues*, which provided a more detailed initiative based on the fundamental pillars of the 2005 platform – legislation and partnership.^11^

Legislation, which is necessary to provide information at the point of consumer decision-making, must be developed from evidence-based research if it is to create a regulatory environment that promotes healthful behavior choices. The success of the European Commission's strategy also depends on partnerships based on voluntary action to increase healthful eating and physical activity levels in the EU. Government agencies of all 27 EU countries work together on the strategy's objectives along with corporations, civil societies, and advocacy organizations. Each organization commits to actions that align with the nutrition strategy of the platform. Since 2005, a total of 300 commitments have been made by partners within the EU.

Another key to the success of partnerships with governments is the ability to target actions at the local level. One of the most successful examples of such a partnership are the EPODE programs and the EPODE European Network (EEN) Project.^12^ Jean-Michel Borys, of the EPODE International Network, NGO, highlighted some of the key points in the methodology that have proven successful in reducing childhood obesity in France. The four pillars for the country's local-level initiatives are as follows: 1) creation of political connections at each level of society, 2) establishment of a sound scientific basis for programs and evaluations, 3) mobilization of resources to establish social marketing, and 4) coordination of a multi-stakeholder approach.

Within each EPODE program, a national coordination team and local project manager coordinate the efforts of stakeholders, nationally and locally. A uniquely effective component of this program is the local steering committee leader, who is appointed by a government official and is responsible for utilizing the local steering committee to engage families at the locations where they receive health information in their communities. Within this framework, in eight pilot towns there was a decrease of almost 10% between 2005 and 2009 in the prevalence of obesity among more than 23,000 children observed.^13^

The EPODE methodology has not only been implemented and accepted throughout France, it is also used in several other countries, including Mexico, the Netherlands, Greece, Spain, Belgium, and Australia. By 2015, the EPODE International Network intends to mobilize key stakeholders in more than 20 countries, support 40 large-scale community-based programs, and involve more than 400 million people worldwide. Though these programs vary from country to country, the basic model is the same: The local mayor initiates the program, and the local project manager, who is hired by the community, implements the program in the field, all with the support of a central coordination team. The family unit is the central piece of the program that leads to healthful behavior change. Stakeholders, including teachers, healthcare professionals, supermarket owners, local media, local non-governmental organizations (NGOs), sport clubs, libraries, local producers, and farmers, foster more healthful environments for families by targeting childhood settings, recreational facilities, socioeconomic policies, and food venues.

To narrow the focus of behavior change down to the individual level, James O. Hill, of the University of Colorado, discussed the techniques people use to prevent weight gain over a lifetime. Dr. Hill presented his analysis, which suggests that people who make a single small change, such as reducing calorie consumption by 100 calories a day, are more likely to prevent weight gain than those who do not make the change.^14^

Intervention trials in both children and adults have shown it is realistic and achievable to make consistent, small reductions in energy intake and similar increases in energy expenditure.^15–18^ Furthermore, in children, such small changes have been shown to diminish excess weight gain. For example, Roderarmel et al.^15^ targeted increasing the number of steps taken daily and eating two servings of breakfast cereal a day (one for breakfast and one as a snack) based on the observation that successful weight maintainers were consistent breakfast eaters. A second study^16^ intervened by increasing the number of daily walking steps and reduced calorie intake by 100 kcal a day by substituting non-caloric sweeteners for sugar. In both studies, the children in the intervention groups who were overweight or obese significantly reduced the trajectory of BMI increase as compared to controls.

*America on the Move*, an organization founded by Dr. Hill to improve health and quality of life by improving healthful eating and active living among individuals, families, communities, and society, uses this small-changes approach as a gateway to a more healthful lifestyle.

Research suggests that obesity spreads through social ties.^19^ These social networks may present an opportunity to create a culture in which social pressure pushes individuals toward more healthful behaviors and overcomes biological and environmental pressures to gain weight. While NCDs are inherently not “communicable,” i.e., transmissible through physical contact, NCDs can be “communicated” diseases, which are contagious through learned behaviors and poor habits. The social nature of behaviors that lead to NCDs must be recognized, as well as the importance of societal norms and peer group behaviors, especially among adolescents, who are at high risk for developing poor health habits. It is essential that health communicators find ways to use social media to encourage adolescents, in their social networks, to adopt more healthful behaviors.

## Confusion about both sides of the energy equation

The increasing prevalence of overweight and obesity around the globe has focused attention on the importance of balancing calories to manage weight. Both the US Centers for Disease Control and Prevention and WHO have stated that overweight and obesity result from an energy imbalance – consuming too many calories combined with inadequate physical activity.^20,21^ Calorie balance is also a key recommendation and communications platform of the recent *Dietary Guidelines for Americans, 2010*.^3^ Nutrition and health communicators are actively seeking strategies that will motivate consumers toward balancing “calories in” with “calories out” to achieve and maintain a healthy weight. Consumer insights are critical to understanding how these concepts are viewed and for creating effective communication strategies.

Laura Fernández Celemín, of the European Food Information Council, presented consumer insights on perceptions of calorie balance within Europe. In-store research in six European countries showed that approximately 70% of consumers are familiar with recommendations to consume fewer calories, yet less than 46% could correctly state their calorie needs.^22^ While many European consumers understand that calorie needs are different for males and females, almost one-third incorrectly believe that children should consume more calories than adults. The majority of European consumers also underestimated calorie expenditure from daily living activities, such as watching television or walking.

Wendy Reinhardt Kapsak, of the IFIC Foundation, provided US consumer insights on energy balance. Several decades of qualitative and quantitative research in the United States suggest a “calorie disconnect.” The Dietary Guidelines Alliance, a US-based, public-private partnership among health societies and industry, in liaison with the federal government, commissioned a qualitative study in 2004 to explore the efficacy of the term “energy balance” in communicating to consumers the concept that calories in must equal calories out. Results from this research suggested that the term “energy balance,” as defined by nutrition and health professional communities, was poorly understood by consumers.^23^

According to the *2011 IFIC Foundation Food & Health Survey*, only 9% of consumers surveyed could accurately estimate their calorie needs per day based on their age, weight, height, and physical activity.^24^ In addition, 69% of Americans did not make an effort to balance calories consumed with calories burned.^24^ Furthermore, while more than half of Americans (57%) perceived themselves as active, less than one of five met the targets outlined in the *2008 Physical Activity Guidelines for Americans*.^25^

The Healthy Weight Commitment Foundation (HWCF), which is supported by almost 200 retailers, food and beverage manufacturers, restaurants, sporting goods retailers and manufacturers, insurance companies, trade associations, NGOs, and professional sports organizations in the United States, is one example of an organization seeking to engage multiple sectors to create effective communication regarding calorie balance and healthy weight. Lisa Gable, of the HWCF, outlined how, in 2009–2011, the foundation focused on three major areas – the workplace, marketplace, and schools.

On May 17, 2010, the HWCF joined First Lady of the United States Michelle Obama and the Partnership for a Healthier America in announcing its pledge to reduce 1.5 trillion calories from the marketplace by the end of 2015, a decrease HWCF member companies intend to sustain in subsequent years. The Robert Wood Johnson Foundation will support a rigorous, independent evaluation and publicly report its findings in the first quarters of 2013 and 2016.

In the workplace arena, food and beverage manufacturers have also made a commitment to increase and improve workplace wellness. The National Business Group on Health is serving as outside evaluator and the first 2-year results, published in 2011,^26^ showed increased availability of wellness programs, including weight management programs, to employees. An additional evaluation will be published in November of 2012 with the third year results.

The HWCF's broad membership is in a unique position to reach out to consumers in the marketplace. While each member develops its own program designed to meet its unique needs and challenges, the foundation serves as a support system and facilitator, when needed, resulting in a more cohesive and comprehensive strategy across a wide range of organizations working toward the same goal. To support their efforts, the HWCF designed the web platforms: http://www.togethercounts.com and http://www.energybalance101.com.

In schools, the HWCF has worked with the Academy of Nutrition and Dietetics and the University of California, Berkeley, employing registered dietitians and physical education instructors to educate students on how to maintain a healthy weight.^27^ The final results of the program are expected to be published at the end of 2013.

Parents and caregivers play a critical role in their children's food intake. In 2010, the Dietary Guidelines Alliance conducted a three-phase consumer research project to determine messages to motivate positive diet and physical activity behaviors among families.^28^ The top-performing consumer messages from this research are outlined in Table 1.

**Table 1:** Top-performing messages for motivating families to lead healthful lifestyles.

Core message	Guidance
Know your number.	Learning how many calories you should consume in a day is a critical first step in managing your weight.
Fun stuff counts as exercise.	Get active with the family, whether it's soccer in the backyard, dancing to music or taking a walk in your neighborhood.
Take charge of your weight.	Balancing the calories you eat and drink with the calories you burn through physical activity puts you in control.
Small steps = big changes.	Serve smaller portions to help curb calories and keep your weight on the right track.
Base your plate on nutrient-rich foods that offer beneficial nutrients and fewer calories.	Choose fruits and vegetables, whole and enriched grains, lean meats, beans and nuts, and low-fat and fat-free dairy foods more often.
You are an important role model for your children.	Show your family how to savor their favorite higher-calorie foods and beverages by enjoying smaller portions together.

Adapted from the Dietary Guidelines Alliance.^28^

Because calorie counting can be confusing, establishing calorie “consciousness” among consumers may be a good place to start a conversation about the role of calories in managing weight. The “know your number” message provides consumers with the individualization they seek, as well as the context they need to make choices, using calorie information provided on food packaging, such as the Nutrition Facts panel (in the United States) and other food labeling initiatives.

Bonnie Taub-Dix, of BTD Nutrition Consultants in New York City, offered realistic and practical insights for helping Americans manage their weight. Regardless of time and place, people must be addressed where they are and not where health communicators wish them to be. Reaching people where they live, work, and play by using communication methods they trust is key to successfully motivating healthful behaviors. In order to succeed, healthful choices must also be easier choices.

The USDA's recently released MyPlate is one example of a simple visual tool to help consumers build a more healthful diet. It is important to communicate not only what is on the plate, but to give clear food examples of what individuals should eat. For example, the communication should be about cheese and milk, instead of “dairy,” or eggs and chicken in lieu of “lean protein.” Finally, families should be encouraged to take time to share meals together. Research has demonstrated that families who eat together have lower rates of drug abuse and cigarette smoking, and more healthful diets, thus contributing to a lower incidence of NCDs.^29^

## Applying effective risk communication to motivate consumers toward healthful lifestyles

Foodborne illnesses are rarely addressed in discussions of NCDs, but an estimated 48 million Americans contract foodborne illnesses each year, with 128,000 being hospitalized and 3,000 dying as a result.^30^ That makes safe food handling practices an important factor in preventing this type of NCD and reducing the economic burden that accompanies it. Robert Gravani, of Cornell University, emphasized how effective risk communication strategies, which are an integral part of addressing food safety and nutrition issues, can be tailored for specific audiences and used to address similar issues related to NCDs.

Best practices for risk communication can be divided into three areas: planning ahead, communicating responsibly, and minimizing harm.^31^ Yet, according to the results of formative focus groups conducted during a Cornell University food safety project, while many consumers can recite the basics of home food safety practices, best practices are often not performed.^32^ To increase consumer compliance, public service announcements (PSAs) using narrative messaging techniques can be developed.

Dr. Gravani presented a case study on a home food safety compliance project that developed and communicated messages about safe food preparation practices through a video PSA project funded by the USDA National Institute of Food and Agriculture.^33^ Focus groups were conducted and PSAs were tested for their effectiveness. Key messages focused on increasing perception of risk, concern for cross-contamination, and basic food safety principles. The “Ugly Bug” PSA video (available at: http://www.fightbac.org/component/content/article/305) was one of the most compelling for consumers.

Risk communication must also be applied during non-crisis times to inform consumers that risks are associated with food and simple actionable steps can be implemented to prevent illness or minimize specific risks through dietary approaches at different life stages. USDA Under Secretary for Food Safety, Elizabeth Hagen, noted that the International Center of Excellence in Food Risk Communication and its website (http://www.foodriskcommunications.org) are important because they provide health professionals around the world with access to the best information available on food risk communications.

In the United States, national multimedia ad campaigns are designed to inform consumers of the risks of improper food-handling practices. For example, in 2011, the USDA, along with the Centers for Disease Control and Prevention and the US Food and Drug Administration, partnered with the Ad Council to create a variety of media components, including television vignettes, print advertisements, radio spots, and web banners, that worked together to demonstrate safe food-handling techniques and prompted consumers to visit http://www.foodsafety.gov for more information on food recalls, food poisoning, and tips for keeping food safe.

European Food Safety Authority (EFSA) Executive Director, Catherine Geslain-Lanéelle, and EFSA Liaison Officer to the US Food and Drug Administration, Jordi Serratosa, emphasized that, in Europe, the EFSA strongly advocates the use of effective risk communication, building on unbiased scientific advice and engaging with key stakeholders along the food chain. The EFSA, which was established in 2002 as Europe's independent risk assessment division, provides scientific opinions that allow EU policy makers to take steps to reduce nutrition-related disease. The EFSA also communicates its findings to a wide range of target audiences and through its website (http://www.efsa.europa.eu) to the public at large.

## Challenges and opportunities for communicating to today's global consumer

IFIC Foundation Chair Nancy Wellman emphasized that reaching consumers through both conventional and social media, as well as multi-stakeholder communication initiatives, is needed to reduce the incidence of NCDs.

Nancy Snyderman, NBC News' Chief Medical Editor, highlighted the critical nature of communicating to different audiences in ways that resonate with each particular community, whether it is via radio, billboards, or mobile device applications. Agents of change should seek out discussions with those whose views do not align with their own, and determine how best to communicate with those who are not currently engaged. This effort would require not only a multisectoral approach that includes public-private partnerships, but also a multifaceted approach, using high- and low-tech efforts.

Speaking about health issues in the simplest terms is critical. To reach target audiences effectively, the words used to communicate public health issues, including NCDs, overweight, and obesity, should be deconstructed and injected with smart science.

Nigel Sunley, of the International Union of Food Science and Technology (IUFoST), which provides a global database of experts on food-related scientific topics for other professional food science bodies to utilize, discussed the importance of communication skills, whether for an individual or an organization. It is critical that scientific information be accurately conveyed to consumers, yet scientists, particularly food scientists, do not always communicate to lay audiences effectively. Food scientists must take on a greater communication role. By providing practical, scientifically accurate information on complex issues, such as genetically engineered foods, and using consumer-friendly language, food scientists can become invaluable food and health communicators.

The larger question is whether countries can find the political will to make the changes necessary to avoid the astronomical economic burden of NCDs. Addressing this issue, Janet Voûte, of Nestlé, representing the International Food and Beverage Alliance (IFBA), described the ongoing debate between NGOs with business interests and NGOs representing public interests. Disagreement exists as to whether both should be at the table during discussions about public health issues. Recently, a group of 137 public interest NGOs signed a letter to the UN Secretary-General asking for clarification on the difference between the two types of organizations. Some argue that business-focused organizations are self-promoting and profit-driven and should be excluded from public health discussions on NCD prevention. Others say public interest NGOs do not have a monopoly on public health concerns. Debates such as these can be polarizing and do not further public health; rather, they may contribute to the public perception that industry is not an appropriate stakeholder in addressing NCDs, overweight, and obesity. If the food industry is being called upon to think creatively about ways to communicate to today's consumers on these health topics, logic dictates that industry representatives should participate in discussions about strategies, best practices, and effective approaches.

There are multiple ways in which the food industry can address health communication challenges. One example, currently in effect, is the commitment through the IFBA of ten leading food and beverage companies to reformulate products; work on clear labeling and nutrition information; restrict marketing of foods high in sugar, salt, and fat to children; promote healthful lifestyles; and work together in partnerships. The IFBA made these commitments to Dr. Margaret Chan, Director-General of WHO, in 2008, and has reported its results annually.

## Simple health communication in practice

To highlight a unique example of NCD prevention in practice, as well as the importance of partnerships and the role of consumer insights, Gustavo Olaiz, of the Mexico Ministry of Health, presented an initiative currently being used in Mexico. The Mexican government, in partnership with industry and NGOs, invited children to teach children about physical activity and health. A special Ministry of Health of Mexico initiative was established – the musical group Amigo de Mí (which translates as “Friend of Myself”) – to promote children's health, nutrition, and exercise. The initiative was further developed by Mexico's First Lady, Lic. Margarita Zavala, by making it inclusive to children with disabilities and ethnic minorities. The children's singing group performs music and exercises to highlight a variety of health and nutrition themes.

Some interim indicators are that the program is having a positive impact. When combined with the music and lyrics, the healthy food plate concept has been remembered correctly by 99% of 25,000 children evaluated. Physical activity has doubled in schools that are using Amigo de Mí from an initial average of 8 minutes per day to more than 15 minutes per day. The results, based on an evaluation of the national program, are expected to be published some time in 2012.

## Conclusion

The IFIC Foundation's daylong Global Diet and Physical Activity Communications Summit, “Insights to Motivate Healthful, Active Lifestyles,” provided expert insights and best practices for the use of science-based, behavior-focused communications in order to motivate individuals and families to achieve healthful, active lifestyles, with the goal of reducing the incidence of NCDs.

Summit Rapporteur Madelyn H. Fernstrom, of the University of Pittsburgh Medical Center, and diet and nutrition editor for NBC's “Today Show,” provided an analysis of the day's proceedings and highlighted major messages that stakeholders should consider as the world faces the growing challenges of NCDs.

As simple, realistic messages are translated from science to practical application, they must be “doable” by the population; the messages most likely to resonate with consumers are simple and realistic. Key messages to the public should be positive and action-oriented and take into consideration where individuals are currently in their lifestyles, and not from where health communicators wish them to be.

Stakeholders should develop a “culture of wellness” and encourage consumers to think about how to obtain a sensible and balanced diet, active lifestyle, and healthy weight. The concept of “energy balance” or “calories in equal calories out” is also key.

Stakeholders must make concerted efforts to teach the public how to evaluate health information, and they must begin health communications early in life. The success of the EPODE program in France demonstrates how connecting with children early in life can benefit the whole family. The personalization of food, nutrition, and health information is of the utmost importance; one approach or recommendation will not work for every individual.

When considering next steps and how to move ahead on NCD prevention and control, stakeholders – including those from government, research and clinical academia, and industry – must become partners, not adversaries. To be successful, health communicators cannot work in silos. Instead, the public and private sectors must work together to develop and deliver consistent messaging.

**Figure 1:**
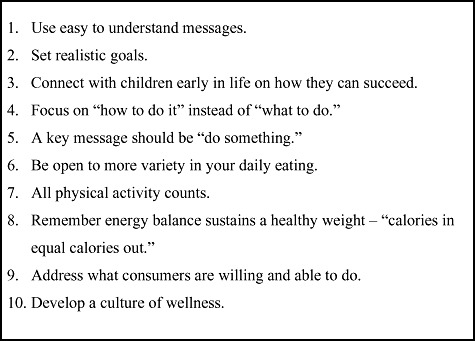
Targeting NCDs: Key messages for communicating with consumers to motivate healthful lifestyles.

Stakeholders must learn from consumers and address the changes that people are both willing and able to make, while creating a culture of wellness tailored to specific groups and their needs. To better communicate information to consumers regarding NCD prevention and control, health communicators should bear in mind the methods and tenets outlined by Dr. Fernstrom in Figure 1.

Amigo de Mí performed songs at the conclusion of the Summit, providing stakeholders with a practical science-based, behavior-focused communications example. The video (in Spanish with English subtitles) is available at http://www.amigodemi.com.mx/videos.php, and the following translated excerpt of the lyrics from the song “Don't Stop” epitomizes both the spirit and the call to action that resulted from the Summit: “Action is always there! And it gets better when you share it. That's why we tell you … from the bottom of our hearts. Don't Stop. You know you have the strength. Don't Stop. Together we will both win!”

## Supporting Information

Appendices: “Communication strategies to help reduce theprevalence of non-communicable diseases,” translated into thesix official languages of the United Nations (Arabic, Chinese,English, French, Russian, and Spanish), include key findings fromthe 2011 International Food Information Council (IFIC) Foundation'sGlobal Diet and Physical Activity Communications Summit:“Insights to Motivate Healthful, ActiveLifestyles.”

Appendix S1: Arabic translation.

Appendix S2: Chinese translation.

Appendix S3: English translation.

Appendix S4: French translation.

Appendix S5: Russian translation.

Appendix S6: Spanish translation.
