# Supplementary material for: Communication strategies to help reduce the prevalence of non-communicable diseases: Proceedings from the inaugural IFIC Foundation Global Diet and Physical Activity Communications Summit
Source: Nutr Rev. 2012 Apr 26;70(5):301–10. doi: 10.1111/j.1753-4887.2012.00480.x (PMC3415678; doi:10.1111/j.1753-4887.2012.00480.x)
Supplement: Supplementary file 1 [file nure0070-0301-SD1.pdf]

INTERNATIONAL
FOOD INFORMATION
COUNCIL FOUNDATION

www.foodinsight.org

دراسات تغذويه - تقرير عن الملتقى (المُزمع عقده في مايو 2012)

نيوترشن ريفيوز (ملاحظات حول التغذية)، المجلد 70، العدد 5
doi: 10.1111/j.1753-4887.2012.00480.x
www.nutritionreviewsjournal.com

نتائج هامة من القمة الدولية للتواصل حول النظام الغذائي والنشاط البدني لمؤسسة مجلس معلومات الغذاء العالمي (IFIC) بعنوان:

"رؤى خاصة بتحفيز نمط حياة نشيطة وصحية"

تركز استراتيجيات التواصل هذه على طرق تخفيف تفشي الأمراض غير المعدية بما في ذلك أمراض القلب والشرايين والسرطان والسكري التي تعزى جزئياً إلى نظام غذائي سيء و قلة النشاط البدني.

استهداف الأمراض الغير معديه : عن طريق التواصل مع المستهلك لتشجيع نمط الحياة الصحية

1. استخدام رسائل سهلة الفهم.
2. وضع أهداف واقعية.
3. التواصل مع الأطفال في مرحلة مبكرة من حياتهم حول كيفية تحقيق النجاح.
4. التركيز على "كيف تفعل" بدلاً من "ماذا تفعل".
5. الرسالة الأساسية يجب أن تكون "افعل شيئاً".
6. حاول أن تزيد من تنوع الأطعمة في وجباتك اليومية.
7. كل الأنشطة البدنيه هامة.
8. تذكر أن التوازن في الطاقة يؤدي إلى وزن صحي –"السعرات الحرارية التي يتم الحصول عليها تساوي السعرات الحرارية التي يتم حرقها".
9. تناول الجوانب التي ينوي المستهلكون فعلها ويقدرون على تحقيقها.
10. تطوير ثقافة الصحة والعافية.

الرسائل الأكثر تأثيراً لتحفيز الأسره لاتباع نمط حياة صحي

الرسالة الأساسية	توجيهات
اعرف عدد السعرات الحراريه.	إن الخطوة الأولى بالغة الأهمية في التحكم في وزنك هي معرفة عدد السعرات الحرارية التي ينبغي عليك استهلاكها في اليوم الواحد.
الأنشطة الممتعة تحسب كتمرينات!	كن نشيطاً مع الأسرة، سواءً كان ذلك بلعب كرة القدم فى ساحة المنزل الخلفية، أو الرقص على أنغام الموسيقى، أو ممارسة رياضة المشي في المنطقة المحيطة بمنزلك.
تحكم في وزنك في وزنك.	إن التوازن بين السعرات الحرارية التي تحصل عليها من الطعام والشراب والسعرات الحرارية التي تقوم بحرقها من خلال النشاط البدنى يجعلك أنت المتحكم في الأمر.
خطوات صغيرة = تغييرات كبيرة.	استهلاك حصص قليلة من الطعام لتقليل السعرات الحرارية والاحتفاظ بوزن صحى.
تناول أطعمة غنية بالعناصر الغذائية بحيث توفر لك العناصر الغذائية المفيدة دون أن تمنحك الكثير من السعرات الحرارية.	أكثر من اختيار الفاكهة والخضروات، الحبوب الكاملة والمدعمة، اللحوم الخالية من الدهن، البقول والمكسرات، منتجات الألبان قليلة الدسم أو الخالية من الدسم.
أنت القدوة الهامه لأطفالك.	اجعل أسرتك تستمتع بمذاق المأكولات والمشروبات ذات السعرات الحرارية العاليه بتناولكم كميات قليله منهاً.

المصدر:دايتري غايدلاينز أليانس ريسيرش 2010 (2010 Dietary Guidelines Alliance Research): تحفيز الأُسر على اتباع أنماط حياة أكثر صحة في 2011 وما بعدها، 2010.
http://www.foodinsight.org/Content/3651/FINAL2010DGAConsumerResearchReport.pdf

لمزيد من المعلومات، يرجى زيارة: www.foodinsight.org/Resources/International-Resources/Global-Summit.aspx
