# Supplementary material for: Communication strategies to help reduce the prevalence of non-communicable diseases: Proceedings from the inaugural IFIC Foundation Global Diet and Physical Activity Communications Summit
Source: Nutr Rev. 2012 Apr 26;70(5):301–10. doi: 10.1111/j.1753-4887.2012.00480.x (PMC3415678; doi:10.1111/j.1753-4887.2012.00480.x)
Supplement: Supplementary file 2 [file nure0070-0301-SD2.pdf]

INTERNATIONAL
FOOD INFORMATION
COUNCIL FOUNDATION

www.foodinsight.org

帮助降低慢性非传染性疾病患病率的沟通策略

来源：*Nutrition Reviews*, Volume 70, Issue 5
doi: 10.1111/j.1753-4887.2012.00480.x
www.nutritionreviewsjournal.com

基于国际食品信息中心（IFIC）基金会的2011全球膳食与身体活动高层研讨会：
“推动积极、健康生活方式的真谛”的主要发现

此交流策略旨在减少包括心血管病、癌症、糖尿病等慢性非传染性疾病的患病率，因为不合理的膳食和身体活动不足是这些疾病的重要病因

针对慢性非传染性疾病：与消费者沟通，推动健康生活方式

1. 运用通俗易懂的信息
2. 设定切实可行的目标
3. 注重儿童的早期生命阶段与成功建立健康生活方式的关系
4. 关注“如何去做”，而不是“做什么”
5. 一个关键的信息是要“有所作为”
6. 每日吃多种食物
7. 所有身体活动都是有意义的
8. 切记只有能量平衡才能维持健康的体重－“能量摄入要与能量消耗平衡”
9. 开展消费者愿意并能够去做的活动
10. 建立一种健康的文化

推动家人养成健康生活方式最要紧的信息

核心信息	指导
知道自己的数字	知道自己每天应该摄取多少能量是控制体重关键的第一步
有趣的活动都可算做身体锻炼！	与家人一起动起来，无论是在后院踢足球，随着音乐舞蹈或是在社区里散步
管控自己的体重	平衡通过饮食摄入的能量与身体活动中消耗的能量就可以掌控自己的体重
小步的积累 = 大改变	减少饭菜分量有助于控制能量的摄入并保持健康体重
选择那些可提供有益营养素且能量较低的食物	更多地选择水果与蔬菜、全谷类和强化谷物、瘦肉、豆类与坚果、低脂和脱脂奶制品
你是你孩子的重要行为榜样	向你的家人示范如何减少所喜爱的高能量食品与饮料的摄入分量

来源：2010 Dietary Guidelines Alliance Research: "Motivating Families to Lead a Healthier Lifestyle in 2011 and Beyond", 2010.
http://www.foodinsight.org/Content/3651/FINAL2010DGAConsumerResearchReport.pdf

了解更多信息，请访问：www.foodinsight.org/Resources/International-Resources/Global-Summit.aspx
