# Supplementary material for: Communication strategies to help reduce the prevalence of non-communicable diseases: Proceedings from the inaugural IFIC Foundation Global Diet and Physical Activity Communications Summit
Source: Nutr Rev. 2012 Apr 26;70(5):301–10. doi: 10.1111/j.1753-4887.2012.00480.x (PMC3415678; doi:10.1111/j.1753-4887.2012.00480.x)
Supplement: Supplementary file 3 [file nure0070-0301-SD3.pdf]

INTERNATIONAL FOOD INFORMATION COUNCIL FOUNDATION

www.foodinsight.org

Communication Strategies to Help Reduce the Prevalence of Non-communicable Diseases

Nutrition Reviews, Volume 70, Issue 5
doi: 10.1111/j.1753-4887.2012.00480.x
www.nutritionreviewsjournal.com

Key findings from the 2011 International Food Information Council (IFIC) Foundation's Global Diet and Physical Activity Communications Summit: "Insights to Motivate Healthful, Active Lifestyles"

These communication strategies focus on ways to reduce the prevalence of non-communicable diseases (NCDs), including cardiovascular disease, cancer, and diabetes, which are attributed, in part, to poor diet and insufficient physical activity.

Targeting NCDs: Communicating with Consumers to Motivate Healthful Lifestyles

1. Use easy to understand messages.
2. Set realistic goals.
3. Connect with children early in life on how they can succeed.
4. Focus on "how to do it" instead of "what to do".
5. A key message should be "do something".
6. Be open to more variety in your daily eating.
7. All physical activity counts.
8. Remember energy balance sustains a healthy weight – "calories in equal calories out".
9. Address what consumers are willing and able to do.
10. Develop a culture of wellness.

Top-Performing Messages for Motivating Families to Lead Healthful Lifestyles

CORE MESSAGE	GUIDANCE
Know your number.	Learning how many calories you should consume in a day is a critical first step in managing your weight.
Fun stuff counts as exercise!	Get active with the family, whether it's soccer in the backyard, dancing to music or taking a walk in your neighborhood.
Take charge of your weight.	Balancing the calories you eat and drink with the calories you burn through physical activity puts you in control.
Small steps = big changes.	Serve smaller portions to help curb calories and keep your weight on the right track.
Base your plate on nutrient-rich foods that offer beneficial nutrients and fewer calories.	Choose fruits and vegetables, whole and enriched grains, lean meats, beans and nuts, and low-fat and fat-free dairy foods more often.
You are an important role model for your children.	Show your family how to savor their favorite higher-calorie foods and beverages by enjoying smaller portions together.

Source: 2010 Dietary Guidelines Alliance Research: "Motivating Families to Lead a Healthier Lifestyle in 2011 and Beyond", 2010.
http://www.foodinsight.org/Content/3651/FINAL2010DGAConsumerResearchReport.pdf

For more information, visit: www.foodinsight.org/Resources/International-Resources/Global-Summit.aspx
