# Supplementary material for: Communication strategies to help reduce the prevalence of non-communicable diseases: Proceedings from the inaugural IFIC Foundation Global Diet and Physical Activity Communications Summit
Source: Nutr Rev. 2012 Apr 26;70(5):301–10. doi: 10.1111/j.1753-4887.2012.00480.x (PMC3415678; doi:10.1111/j.1753-4887.2012.00480.x)
Supplement: Supplementary file 4 [file nure0070-0301-SD4.pdf]

INTERNATIONAL
FOOD INFORMATION
COUNCIL FOUNDATION

www.foodinsight.org

Stratégies de Communication pour la Réduction de la Prévalence des Maladies non Transmissibles

Nutrition Reviews, Volume 70, Numéro 5
doi: 10.1111/j.1753-4887.2012.00480.x
www.nutritionreviewsjournal.com

Principales conclusions du sommet mondial organisé par l'"International Food Information Council (IFIC) Foundation" au sujet de la Communication sur l'Alimentation et l'Activité Physique: "Insights to Motivate Healthful, Active Lifestyles"

Ces stratégies de communication se concentrent sur les moyens de réduire la prévalence des maladies non transmissibles, en particulier les maladies cardio-vasculaires, le cancer et le diabète qui sont en partie attribués à une alimentation non adaptée et à un manque d'activité physique.

Cibler les Maladies non Transmissibles: Communiquer avec les Consommateurs pour Stimuler un Mode de Vie plus Sain

1. Utiliser des messages faciles à comprendre.
2. Définir des objectifs réalistes.
3. Eduquer les enfants le plus tôt possible sur les moyens d'y parvenir.
4. Insister sur "comment le faire" plutôt que "quoi faire".
5. Le message clé devrait être "faites quelque chose".
6. Etre ouvert à une alimentation quotidienne plus variée.
7. Toute activité physique joue un rôle.
8. Se souvenir qu'un bon équilibre de l'énergie est l'assurance d'un poids équilibré – "l'ingestion de calories doit être contrebalancée par les dépenses de calories".
9. Se préoccuper de ce que les consommateurs sont prêts à faire et sont capables de faire.
10. Développer une culture du bien-être.

Messages les plus Efficaces pour Motiver les Familles à Adopter un Mode de Vie plus Sain

MESSAGES PRINCIPAUX	CONSEILS
Connaître le bon nombre.	Apprendre le nombre de calories à consommer chaque jour est une première étape essentielle pour la gestion de votre poids.
S'amuser, c'est aussi se dépenser!	Dépensez-vous avec votre famille, que ce soit en jouant au football dans la cour, en allant danser ou en faisant un tour à pied dans le quartier.
Prendre son poids en main.	Eliminer les calories ingérées par la nourriture et les boissons en pratiquant une activité physique vous aide à contrôler votre poids.
Petites étapes = gros changements.	Servez de petites portions pour limiter le nombre de calories et pour garder votre poids sur la bonne voie.
Garnissez votre assiette d'une nourriture qui offre des nutriments bénéfiques et qui soit pauvre en calories.	Choisissez plus souvent des fruits et des légumes, des céréales complètes et enrichies, des viandes maigres, des haricots et des noix, ainsi que des produits laitiers allégés en matières grasses.
Vous représentez un modèle important pour vos enfants.	Montrez à votre famille comment profiter de leurs plats et de leurs boissons favoris riches en calories, en savourant ensemble de plus petites portions.

Source: 2010 Dietary Guidelines Alliance Research: "Motivating Families to Lead a Healthier Lifestyle in 2011 and Beyond", 2010.
http://www.foodinsight.org/Content/3651/FINAL2010DGAConsumerResearchReport.pdf

Pour plus d'informations, visitez www.foodinsight.org/Resources/International-Resources/Global-Summit.aspx
