# Supplementary material for: Communication strategies to help reduce the prevalence of non-communicable diseases: Proceedings from the inaugural IFIC Foundation Global Diet and Physical Activity Communications Summit
Source: Nutr Rev. 2012 Apr 26;70(5):301–10. doi: 10.1111/j.1753-4887.2012.00480.x (PMC3415678; doi:10.1111/j.1753-4887.2012.00480.x)
Supplement: Supplementary file 5 [file nure0070-0301-SD5.pdf]

INTERNATIONAL
FOOD INFORMATION
COUNCIL FOUNDATION

www.foodinsight.org

Стратегии коммуникации для помощи в снижении распространенности неинфекционных заболеваний (НИЗ)

Nutrition Reviews, Volume 70, Issue 5
doi: 10.1111/j.1753-4887.2012.00480.x
www.nutritionreviewsjournal.com

Основные выводы Совещания на высшем уровне 2011 г. по глобальному питанию и физической активности Международного Фонда IFIC: "Принципы стимулирования здорового, активного образа жизни"

Данные стратегии коммуникации делают упор на путях сокращения распространенности неинфекционных заболеваний (НИЗ), включая сердечно-сосудистые заболевания, рак и диабет, которые связаны, в первую очередь, с неправильным питанием и недостаточной физической активностью.

Выбор НИЗ: Связь с потребителями с целью мотивации здорового образа жизни

1. Используйте легкие для понимания обращения.
2. Устанавливайте реальные цели.
3. Общайтесь с маленькими детьми на тему, как они могут достичь успеха.
4. Сосредотачивайтесь на том "как сделать" вместо того "что делать".
5. Ключевым обращением должно быть "Сделай что-нибудь".
6. Разнообразьте Ваше ежедневное питание.
7. Учитывается любая физическая активность.
8. Помните, что энергетический баланс поддерживает массу тела в норме – "сколько калорий пришло, столько должно уйти".
9. Проявляйте внимательное отношение к тому, что потребители желают и в состоянии делать.
10. Разрабатывайте культуру оздоровления.

Самые результативные обращения для мотивации семей к ведению здорового образа жизни

ОСНОВНАЯ ИДЕЯ	РУКОВОДСТВО
Знай свое число.	Знание того, сколько калорий тебе следует потребить в течение дня, является первым важным шагом в осуществлении контроля над весом.
Забавы считаются физическими упражнениями!	Веди с семьей активный образ жизни, неважно, или это футбол во дворе, танцы под музыку или прогулка по своему району.
Контролируйте свой вес.	Поддержание баланса между калориями, потребляемыми с пищей и теми, которые вы сжигаете при физической активности заставляет вас следить за собой.
Небольшие шаги = большие изменения.	Делайте небольшие порции, чтобы ограничить поступление калорий и поддерживать правильный вес.
Положите в основу своего рациона питательные продукты, содержащие полезные нутриенты и меньшее количество калорий.	Чаще выбирайте фрукты и овощи, цельнозерновой и обогащенный хлеб, нежирное мясо, бобовые и орехи, нежирные или обезжиренные молочные продукты.
Вы выполняете важную роль примера для своих детей.	Покажите членам Вашей семьи, как смаковать небольшими порциями их любимые высококалорийные продукты и напитки.

Источник: Исследования Руководства по питанию 2010: "Мотивации семей к ведению здорового образа жизни в 2011 и последующий период", 2010.
http://www.foodinsight.org/Content/3651/FINAL2010DGAConsumerResearchReport.pdf

Для получения дополнительной информации посетите сайт
www.foodinsight.org/Resources/International-Resources/Global-Summit.aspx
