# Supplementary material for: Communication strategies to help reduce the prevalence of non-communicable diseases: Proceedings from the inaugural IFIC Foundation Global Diet and Physical Activity Communications Summit
Source: Nutr Rev. 2012 Apr 26;70(5):301–10. doi: 10.1111/j.1753-4887.2012.00480.x (PMC3415678; doi:10.1111/j.1753-4887.2012.00480.x)
Supplement: Supplementary file 6 [file nure0070-0301-SD6.pdf]

INTERNATIONAL
FOOD INFORMATION
COUNCIL FOUNDATION

www.foodinsight.org

Estrategias comunicacionales para ayudar a reducir la prevalencia de enfermedades no transmisibles

Nutrition Reviews, volumen 70, N°5
doi: 10.1111/j.1753-4887.2012.00480.x
www.nutritionreviewsjournal.com

Resultados clave de la Cumbre Mundial 2011 sobre Alimentación y Actividad Física de la Fundación IFIC: "Percepciones para motivar estilos de vida activos y saludables"

Estas estrategias comunicacionales se centran en las formas para reducir la prevalencia de enfermedades no transmisibles, incluidas la enfermedad cardiovascular, el cáncer y la diabetes, que son atribuidas, en parte, a una dieta pobre y la falta de actividad física.

Objetivos para enfrentar las enfermedades no transmisibles: comunicación con los consumidores para motivar la implementación de estilos de vida saludables

1. Utilizar mensajes fáciles de comprender.
2. Establecer metas realistas.
3. Conectarse con los niños a temprana edad y hablar sobre el modo en que pueden tener éxito para tener una vida sana.
4. Centrarse en "cómo hacerlo" en vez de "qué hacer".
5. Un mensaje clave debería ser "haga algo".
6. Estar abierto a una variedad más amplia de alimentos en su alimentación diaria.
7. Toda la actividad física cuenta.
8. Recordar que el equilibrio energético mantiene un peso saludable: "las calorías que entran deben igualar a las calorías que salen".
9. Tomar en cuenta lo que los consumidores estén dispuestos y sean capaces de hacer.
10. Desarrollar una cultura del bienestar.

Mensajes que más resultan para motivar a las familias a llevar estilos de vida saludables

MENSAJE CENTRAL	PAUTA
Conozca su número.	Aprender cuántas calorías debería consumir por día es un importante primer paso en el manejo de su peso.
¡Las cosas divertidas cuentan como ejercicio!	Mántengase activo con su familia, ya sea jugando fútbol en el patio, bailando o dando un paseo por el barrio.
Hágase cargo de su peso.	Equilibrar las calorías que come o bebe con las calorías que quema a través de la actividad física lo hacen tener el control.
Pequeños pasos = grandes cambios.	Sirva porciones más pequeñas para ayudar a poner freno a las calorías y mantener su peso por el camino correcto.
Base sus platos en alimentos ricos en nutrientes que sean beneficiosos y con menos calorías.	Escoja con más frecuencia frutas y verduras, granos enteros enriquecidos, carnes magras, frijoles y nueces, y lácteos bajos o libres de grasas.
Usted es un modelo importante para sus hijos.	Muestre a su familia cómo saborear sus alimentos y bebidas favoritos que contienen más calorías, disfrutando juntos de porciones más pequeñas.

Fuente: 2010 Dietary Guidelines Alliance Research: "Motivating Families to Lead a Healthier Lifestyle in 2011 and Beyond", 2010.
http://www.foodinsight.org/Content/3651/FINAL2010DGAConsumerResearchReport.pdf

Para mayor información, visite la página Web www.foodinsight.org/Resources/International-Resources/Global-Summit.aspx
